# Testing and Treating the Missing Millions with Tuberculosis

**DOI:** 10.1371/journal.pmed.1001805

**Published:** 2015-03-24

**Authors:** Madhukar Pai, Puneet Dewan

**Affiliations:** 1 McGill Global Health Programs & McGill International TB Centre, McGill University, Quebec, Canada; 2 Bill & Melinda Gates Foundation, New Delhi, India

## Abstract

In a Guest Editorial on World TB Day, Madhukar Pai and Puneet Dewan identify programmatic and policy changes needed to end TB by 2035.

The Stop TB Partnership’s theme for World Tuberculosis Day 2015, “Reach, Treat, Cure Everyone,” calls for a global effort to find and definitively treat all people with tuberculosis (TB) and accelerate progress towards the ambitious goal of ending TB by 2035 [1].

Every year, among the estimated 9 million persons who develop TB, 3 million are missed—not diagnosed, treated, nor notified to national TB programs [2]. Reaching all these individuals and ensuring accountable, effective TB treatment will require TB control programs to adopt innovative tools and modernize program service delivery.

TB control programs are beginning to retool and adopt new, molecular diagnostics that are more sensitive than smear microscopy and capable of rapidly detecting drug resistance [3]. For example, over 10 million Xpert MTB/RIF (Cepheid Inc, Sunnyvale, CA, USA) tests have been used in 116 countries [4]. With more laboratories offering rapid molecular tests, there has been a 3-fold increase in the number of multidrug-resistant TB (MDR-TB) cases detected [2]. The “Test and Treat” strategy, where everyone diagnosed with active TB is offered a drug susceptibility test to guide choice of treatment, is a key element of the “End TB Strategy” [1] and is becoming the standard of care in many settings [5,6].

Along with new diagnostics, retooling is also needed to prepare for new drug regimens. With the introduction of bedaquiline, delamanid, and the expected introduction of a new drug regimen called PaMZ, consisting of pretomanid (previously called PA-824), moxifloxacin, and pyrazinamide, the TB community is gearing up for the introduction of new, shorter treatment regimens within the next five years. To secure the benefits of the new regimens, national TB programs will need to streamline regulatory and policy adoption processes and coordinate introduction of new treatment protocols, along with companion diagnostics [7].

In order to ensure that new tools reach those who need them, new models are needed to engage informal (i.e., unqualified) and private sector health care providers who dominate the health care marketplace in highly privatized health markets [8]. National disease control programs focused on public sector health services have left these frontline care providers severely underutilized in TB control.

Serving the unreached millions of people with TB will require innovative service delivery models. Social franchises, social enterprises, insurance-based initiatives, intermediary agencies, and provider aggregation models all offer new paths to engage, educate, and incentivize underutilized care providers to offer quality and affordable TB care. Several countries, including India, Pakistan, Bangladesh, and Indonesia are experimenting with such models [9,10].

While waiting for better therapies, TB programs can increase their effectiveness by improving treatment adherence and harnessing the transformative potential of information and communication technology for eHealth services. For example, directly observed therapy (DOT) has, for decades, been an important means to monitor treatment adherence and remains trusted and widely used by public health programs, despite an inconclusive evidence base [11]. In the meantime, the number of mobile phone connections is rapidly approaching the number of people in many lower- and middle-income countries, presenting an opportunity to optimize electronic monitoring of medication adherence in programmatic settings. eHealth innovations can have synergistic impact with improved diagnostics and drug regimens by empowering health care workers to recognize and address attrition along the pathway of seeking health care.

In fact, India is currently experimenting with innovative projects in which private doctors use mobile phones to call a contact center to notify TB cases and ensure that their patients receive e-vouchers for free and appropriate drugs [12]. In just the first few months, private providers have enthusiastically responded and initiated treatment for thousands of patients through these initiatives, and call center operators are actively pushing information on care gaps to patients and providers and facilitating treatment adherence monitoring. These innovative approaches are bringing previously unreported, privately-treated TB patients into the light of public health services, where care and adherence can be monitored. These emerging models are expected to reduce costs to patients and improve adherence and outcomes [12].

None of the above strategies can be implemented at scale without adequate funding by donors, the private sector, and, most importantly, governments. In the long run, TB control will bring significant human, economic, and health benefits to any country [13]. While some countries, such as China, have recognized this, increased their investments in TB control, and shown substantial reductions in disease prevalence [14], other countries continue to lag behind. For example, despite announcing an ambitious plan to dramatically intensify TB control by 2020 [15], India has cut back on both health budgets in general and TB spending in particular [16].

Political leaders and policy makers need to understand that TB cannot be eliminated without investing more resources. Here, advocacy is critical.

There are signs that TB’s time in the spotlight is arriving. The United Nations Secretary-General recently appointed Eric Goosby of the United States as the new UN Special Envoy on TB, while actress Emma Thompson stepped forward to serve as a TB ambassador in England. In India, TB came out of the shadows recently, when one of India’s most celebrated superstars, Amitabh Bachchan, publicly announced that he was a TB survivor and assumed his new role as TB Ambassador for Mumbai Mission for Tuberculosis Control [12]. Recently, one of India’s most popular TV shows, “Satyamev Jayate,” focused an entire episode on TB, bringing unprecedented attention to the disease [12].

These advocates and champions are bringing much needed public and political attention to a disease that has been neglected for far too long. We hope such advocacy will enable national TB programs to argue for and get the resources they need to modernize TB control and reach the missing millions.

## Abbreviations:

DOT: directly observed therapy
MDR-TB: multidrug-resistant tuberculosis
TB: tuberculosis
